# Resilience Factors That Modify Genetic Risk for ADHD Matter for Later Life Emotional and Cognitive Health Outcomes

**DOI:** 10.1093/geroni/igaa057.1484

**Published:** 2020-12-16

**Authors:** Thalida Arpawong, Joel Milam

**Affiliations:** 1 University of Southern California, Claremont, California, United States; 2 University of Southern California, Los Angeles, California, United States

## Abstract

Having features of Attention Deficit Hyperactivity Disorder (ADHD) is associated with challenges with emotional regulation and cognitive function. Heritability for ADHD in adults is estimated to be 30%. The degree to which genetic risk for ADHD can be modified by protective factors, such as strong personal relationships and pursuing more education, to result in better emotional and cognitive outcomes at later ages is not well understood. We evaluated these relationships in a population-representative sample of older adults in the U.S. Health and Retirement Study, with 9,003 European Americans (EA; 57% women, age M=68.6, SD=10.4), and 1,622 African Americans (AA; 63% women, age M=64.4, SD=9.5). Outcomes included validated scales for psychological resilience, life satisfaction, depressive symptoms (DepSx), cognitive functioning, and impairment, assessed between 2008-2012. A genetic risk score for ADHD (GRS-ADHD) was calculated from a genomewide-scan, using a mixed ancestry sample. We used multivariable linear and logistic regression models, adjusted for age, gender, and genetic ancestry. We found a protective effect such that stronger personal relationships in adulthood reduced the inverse relationship between the GRS-ADHD and resilience and life satisfaction in later age (interaction p’s<.004 in EAs), but not with DepSx. In contrast, strong parental relationships in childhood attenuated the association between the GRS-ADHD and later life DepSx (interaction p’s<.007 in EAs and AAs) only. Education did not modify, but mediated the main effect of the GRS-ADHD on cognitive abilities and impairment in EAs and AAs. Findings have implications for later age health for those at greater genetic risk for ADHD.

